# A case of eosinophilic bronchiolitis after the initiation of immune checkpoint inhibitor

**DOI:** 10.1111/1759-7714.14931

**Published:** 2023-05-18

**Authors:** Akiko Tamura, Masao Hashimoto, Atsuko Hosoi, Masayuki Hojo

**Affiliations:** ^1^ National Center for Global Health and Medicine Hospital, Respiratory Medicine Tokyo Japan; ^2^ National Center for Global Health and Medicine Hospital, Pathology Tokyo Japan

**Keywords:** atezolizumab, bronchiolitis, eosinophil, immune checkpoint inhibitor, immune‐related adverse event

## Abstract

A 50‐year‐old Japanese woman with advanced breast cancer presented with productive cough and dyspnea while she was receiving a sixth cycle of chemotherapy including atezolizumab. Chest computed tomography revealed bronchiolitis and transbronchial lung cryobiopsy revealed eosinophilic bronchiolitis. Corticosteroid therapy successfully resolved her symptoms. Eosinophilic bronchiolitis is a rare but important immune‐related adverse event; herein, we discuss its diagnosis and possible pathophysiology.

## Graphical abstract

A 50‐year‐old Japanese woman with advanced breast cancer presented with productive cough and dyspnea while she was receiving chemotherapy containing atezolizumab. From chest computed tomography and transbronchial lung cryobiopsy, she was diagnosed with eosinophilic bronchiolitis, and corticosteroid therapy successfully resolved her symptoms. Eosinophilic bronchiolitis is a rare but important immune‐related adverse event; herein, we discuss its diagnosis and possible pathophysiology.

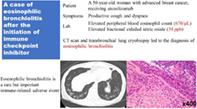

## INTRODUCTION

Immune checkpoint inhibitors (ICIs) have led to breakthroughs in cancer therapy, and their indications have been expanding to various cancer types. However, the growing attention on ICIs has shed light on immune‐related adverse events (irAEs). Pulmonary complications of irAEs are reported in 4% of ICI treatments, mostly interstitial lung diseases (ILDs); airway diseases are rarely reported.^1^, ^2^ Herein, we report a case of eosinophilic bronchiolitis after treatment with atezolizumab, a programmed cell death ligand 1 (PD‐L1) inhibitor, and discuss its possible pathophysiology from the perspective of the relationship between PD‐L1/PD‐L2 expression and T‐helper (Th)1, Th2, and Th17 cells.

## CASE REPORT

A 50‐year‐old Japanese woman with hormone receptor‐positive and HER2 negative, stage IV breast cancer, who was receiving systemic anticancer therapy containing atezolizumab, bevacizumab, and paclitaxel, presented with productive cough and dyspnea, which started around the beginning of the sixth cycle and progressively worsened during the course of treatment. Her other medical history included smoking cessation at 5‐pack‐year, allergic rhinitis, and sinusitis, for which she took antihistamines. She also had asthma; however, inhaled corticosteroids were not prescribed because the latest exacerbations had occurred 20 years prior and she had not experienced any further asthma attacks.

**FIGURE 1:**
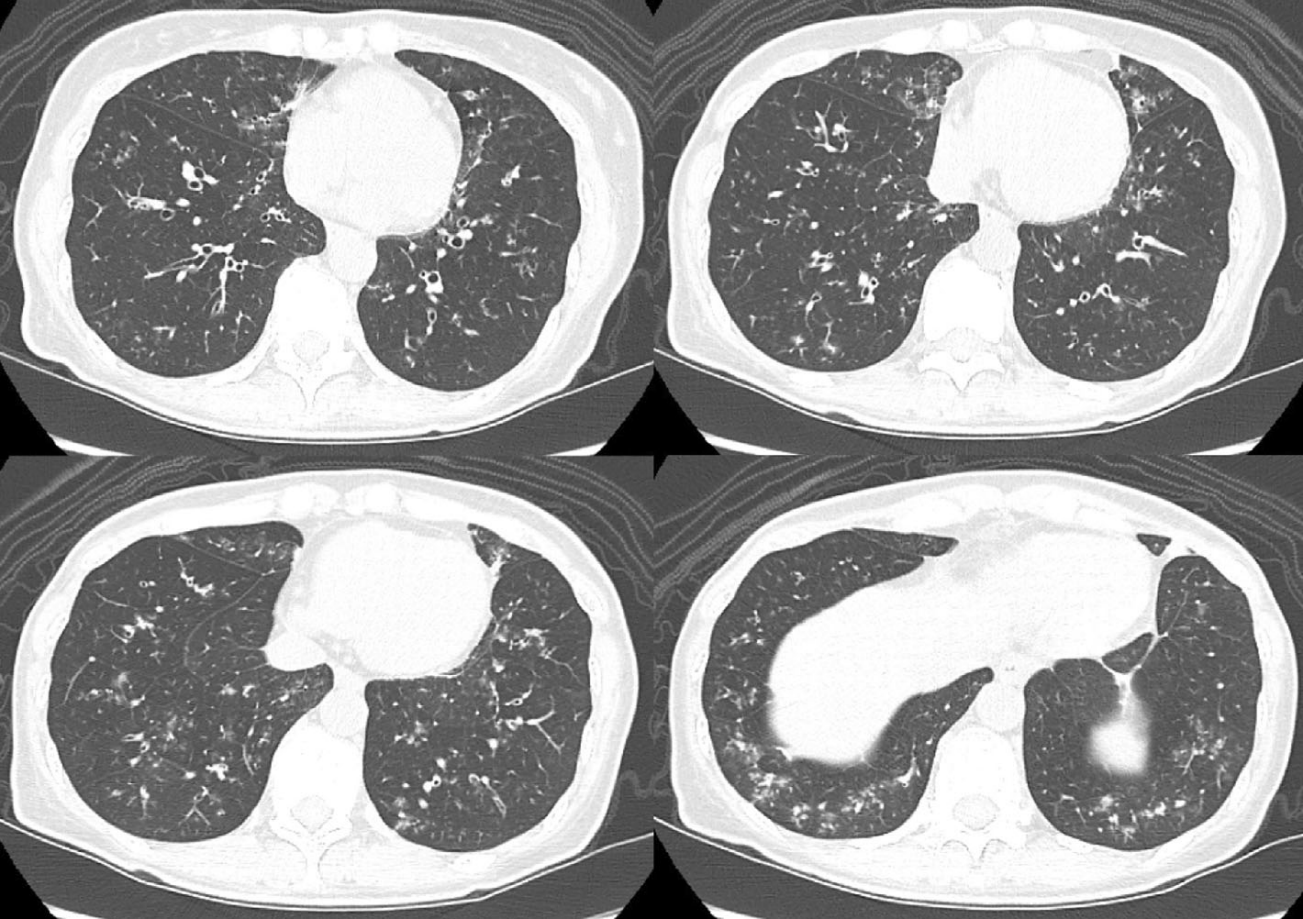
Chest computed tomography (CT) taken after symptom onset, revealing bronchiolitis.

On arrival, she was febrile with a body temperature of 38.0°C, respiratory rate of 18/min and oxygen saturation of 96% at room air. Her lung sounds were clear without wheezing. Laboratory findings revealed a white blood cell count of 8700/μL with 7.7% eosinophil, and C‐reactive protein of 1.32 mg/dL. The total IgE level was 201 IU/mL. From the sputum culture test, only normal flora, such as α‐streptococcus and γ‐streptococcus, was isolated. The sputum cytology revealed mostly neutrophils, followed by eosinophils and lymphocytes. Pulmonary function tests revealed mixed obstructive and restrictive ventilatory defects (forced expiratory volume in 1 s [FEV_1_] of 1.46 L, forced vital capacity [FVC] of 2.40 L, FEV_1_ to FVC ratio [FEV_1_/FVC] of 60.8%, and vital capacity to average ratio [%VC] of 78%), without responsiveness to bronchodilators. Her fractional exhaled nitric oxide (FeNO) level was mildly elevated at 34 ppb. Chest computed tomography (CT) revealed thickening of the bronchial wall and a diffuse tree‐in‐bud appearance, consistent with bronchiolitis, which had not been seen on the chest CT prior to anticancer therapy (Figure 1). Antinuclear antibodies, antineutrophil cytoplasmic antibodies, and cyclic citrullinated peptide antibodies were tested for suspected connective tissue disease‐related bronchiolitis, but all were negative. Human leukocyte antigen and human T lymphotropic virus type 1 (HTLV‐1) antibody tests were also performed for suspected sinobronchial syndrome and HTLV‐1 associated bronchioloalveolar disorder, respectively. However, none of the examinations provided results supporting these diagnoses.

**FIGURE 2:**
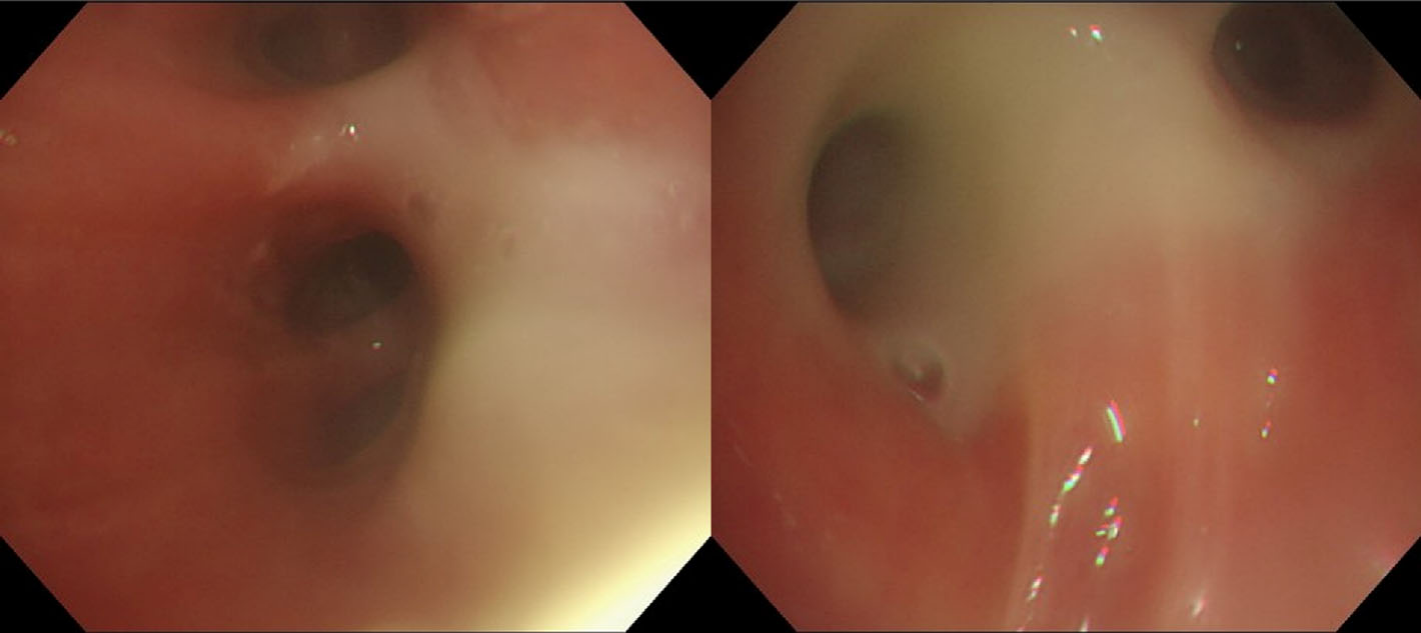
Bronchoscopic examination revealed an edematous bronchial wall filled with purulent sputum.

She initially received ampicillin‐sulbactam and low‐dose clarithromycin; however, her symptoms and CT findings did not improve. Long‐acting beta‐agonist was also ineffective; inhaled corticosteroids were not initiated since the possibility of infection had not been completely ruled out. She then underwent bronchoscopy which revealed edematous mucosa filled with purulent sputum (Figure 2). The cytology of sputum collected by suctioning with bronchoscopy showed abundant inflammatory cells, largely neutrophils, followed by eosinophils; however, the sputum was sterile. Although few lesions were seen in the right middle lobe and lingula on the CT scan, bronchoalveolar lavage was carried out on the medial segmental bronchus of the right middle lobe, which revealed 45% neutrophils, 42% macrophages, 12% lymphocytes, and 1% eosinophils.

**FIGURE 3:**
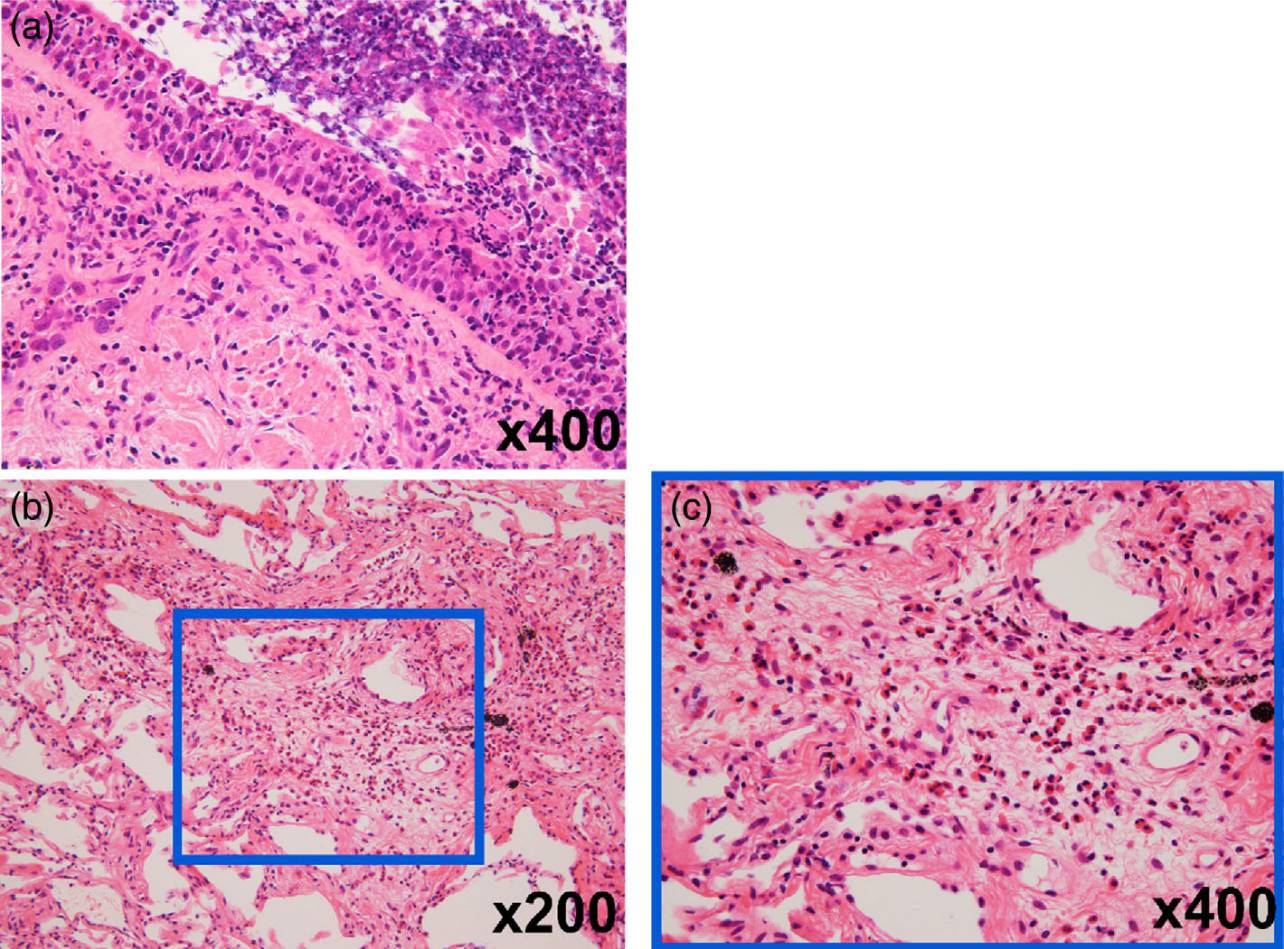
Microscopic findings of transbronchial lung cryobiopsy. (a) Infiltration of various inflammatory cells is observed in the bronchial epithelium. (b, c) An increase in eosinophils is observed in the stroma surrounding the bronchioles.

Transbronchial lung cryobiopsy was also performed at the lateral branch of the right anterior basal segmental bronchus (B8a) and of the right lateral basal segmental bronchus (B9a), with a probe diameter of 1.9 mm and freezing time of 5 s. The results revealed infiltration of various inflammatory cells in the bronchioles and bronchi in which eosinophils were predominant, especially in the stroma around the bronchioles (Figure 3). In contrast, the alveoli remained relatively intact. No typical characteristics of asthma, such as goblet cell hyperplasia, or diffuse panbronchiolitis, such as lymphocyte‐dominant inflammation, were observed. Finally, neither lymphoid follicle hyperplasia nor bronchiole fibrosis were observed, thereby reducing the likelihood of follicular or obstructive bronchiolitis.

**FIGURE 4:**
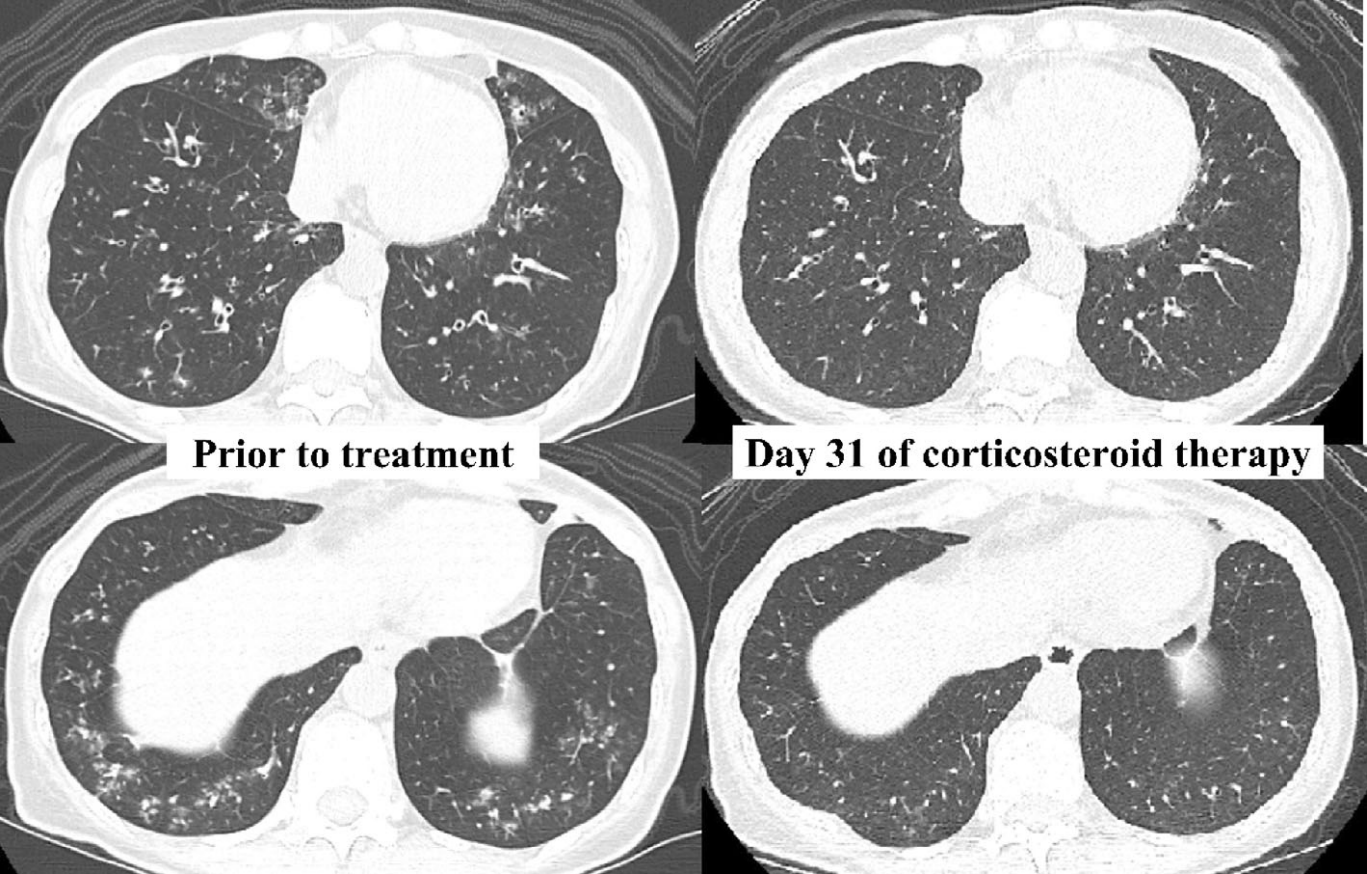
Chest computed tomography (CT) prior to (on the left side) and 31 days after (on the right side) the corticosteroid therapy revealing the improvement of eosinophilic bronchiolitis.

Therefore, she was diagnosed with eosinophilic bronchiolitis and was prescribed 50 mg/day of prednisolone, based on grade 2 irAE of pneumonitis. Her symptoms improved rapidly, and a CT scan performed on day 8 of steroid treatment revealed improvement in bronchiolitis. On day 31, follow‐up CT showed resolution of most of the pulmonary lesions, and her pulmonary function also improved (FEV_1_, 2.19 L; FEV_1_/FVC, 73.7%; and %VC, 85.7%). Prednisolone was successfully reduced to 20 mg/day (Figure 4).

## DISCUSSION

To the best of our knowledge, this is the first reported case of eosinophilic bronchiolitis occurring after the initiation of ICI therapy. Peripheral blood eosinophil counts and FeNO served as clues to eosinophil activation, and a biopsy led to a diagnosis. Pulmonary irAEs largely consist of ILDs, such as organizing pneumonia, and the incidence of airway disease is mostly limited to individual case reports.^3^, ^4^ Additionally, regardless of the use of ICIs, eosinophilic bronchiolitis itself is a rare disease in which patients experience dyspnea and productive cough with eosinophilia in the bronchioles.^5^, ^6^ A literature review of seven patients showed that all patients were Asian, five had asthma, and three had sinusitis.^6^ Our patient shared common features with these patients, indicating that she may have been susceptible to eosinophilic bronchiolitis and that the use of an ICI triggered its development.

**FIGURE 5:**
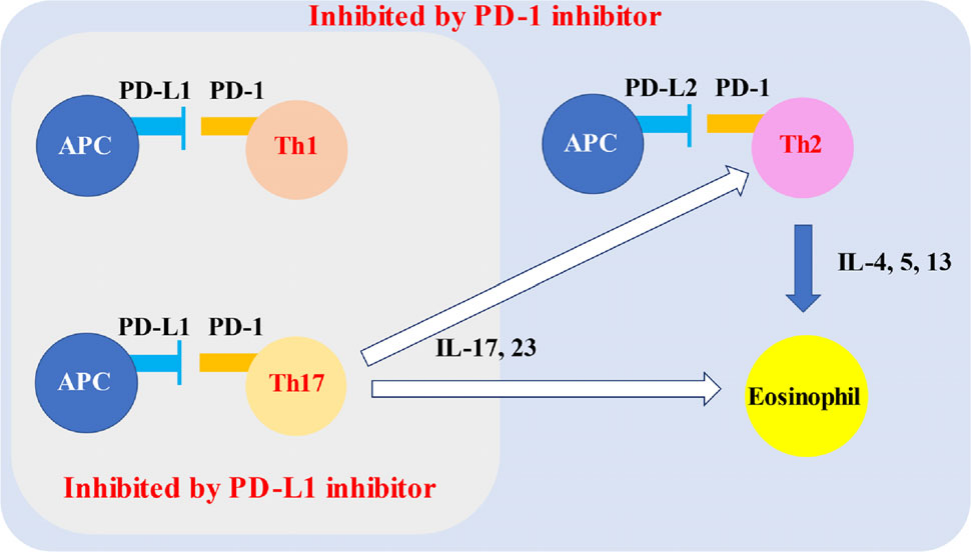
Relationship between PD‐L1/PD‐L2 expression and Th1, 2, 17 activations. Through PD‐1/PD‐L1 or PD‐1/PD‐L2 interaction, antigen presenting cells suppress helper T cell activation and cytokine production. In the presence of PD‐1 inhibitor, these interactions are inhibited. PD‐L1 inhibitor also suppresses PD‐1/PD‐L1 interaction, but not PD‐1/PD‐L1 interaction. The hypothesized link between PD‐L1 inhibitor and eosinophil activation is that Th17 produces cytokines which stimulate Th2 and eosinophils. IL, interleukin; PD‐1, programmed cell death‐1; PD‐L1, programmed cell death‐1 ligand 1; PD‐L2, programmed cell death‐1 ligand 2; Th, T‐helper.

From the molecular perspective of irAEs, it has been suggested that differences in the expression of PD‐L1 and PD‐L2 in antigen‐presenting cells affect the balance of Th1, Th2, and Th17 inflammation (Figure 5).^7^, ^8^ In murine models, the blockage of PD‐L1 enhances Th1 and Th17 inflammation, whereas the blockage of PD‐L2 enhances Th2 inflammation.^7^ Previous reports on other lung‐related eosinophilic irAEs, such as asthma and eosinophilic pneumonia, were mostly due to PD‐1 inhibitors, which block the interaction between PD‐1 and PD‐L2, as well as PD‐1 and PD‐L1.^9^, ^10^, ^11^ The inhibition of PD‐L2 signaling enhances the activity of Th2 cells, which produce interleukin (IL)‐4, IL‐5, and IL‐13 and facilitate eosinophil recruitment, leading to the development of asthma and eosinophilic pneumonia.^8^, ^9^ However, this explanation was not applicable to our patient as she received atezolizumab, a PD‐L1 inhibitor that blocks the interaction between PD‐1 and PD‐L1, and B7.1 and PD‐L1, but not PD‐1 and PD‐L2. One possible explanation is that Th17 cells activate eosinophils by releasing IL‐17 and IL‐23.^12^, ^13^ IL‐17 and IL‐5 collectively enhance eosinophil activity, in which IL‐5 contributes to eosinophil survival and IL‐17 causes eosinophil degranulation by interacting with IL‐17 receptors expressed on eosinophils.^12^ Additionally, IL‐23 is shown to upregulate Th2 mediated eosinophil recruitment into airways.^13^ Th17 cells also activate other immune cells, such as neutrophils and macrophages, which may explain the heterogeneity of inflammatory cell infiltration into the epithelium.^13^

In conclusion, eosinophilic bronchiolitis is a rare but important irAE. Peripheral blood eosinophil counts and FeNO levels should be measured to evaluate eosinophil activity. Prompt treatment with steroids will benefit patients through the rapid relief of symptoms and avoidance of delays in cancer treatment.

## AUTHOR CONTRIBUTIONS

Akiko Tamura collected data of this clinical case and wrote the manuscript with support from co‐authors. Masao Hashimoto provide conceptual ideas of case report, and the proof outline. Atsuko Hosoi interpreting result of pathology. Masayuki Hojo supervised the manuscripting of this case report. All authors discussed the interplitation of this clinical case and commented on the manuscript.

## CONFLICT OF INTEREST STATEMENT

The authors declare that they have no conflicts of Interest.

## PATIENT CONSENT FOR PUBLICATION

Written informed consent was obtained from the patient for publication of this case report and accompanying images.
